# LncRNA NONRATT021972 siRNA regulates neuropathic pain behaviors in type 2 diabetic rats through the P2X_7_ receptor in dorsal root ganglia

**DOI:** 10.1186/s13041-016-0226-2

**Published:** 2016-04-23

**Authors:** Shuangmei Liu, Lifang Zou, Jinyan Xie, Wei Xie, Shiyao Wen, Qiuyu Xie, Yun Gao, Guilin Li, Chunping Zhang, Changshui Xu, Hong Xu, Bing Wu, Qiulan Lv, Xi Zhang, Shouyu Wang, Yun Xue, Shangdong Liang

**Affiliations:** Department of Physiology, Medical College of Nanchang University, Nanchang, Jiangxi 330006 People’s Republic of China; Clinic Medicine Department, Undergraduate Student of Grade 2012, Medical College of Nanchang University, Nanchang, Jiangxi 330006 People’s Republic of China; Department of Cell Biology, Medical College of Nanchang University, Nanchang, Jiangxi 330006 People’s Republic of China

**Keywords:** P2X_7_ receptor, Long noncoding RNA, Diabetic neuropathic pain, Dorsal root ganglia

## Abstract

**Background:**

Long non-protein-coding RNAs (lncRNAs) are involved in the pathological processes of nervous system diseases. NONRATT021972 is an lncRNA. This study explores the effects of lncRNA NONRATT021972 small interference RNA (siRNA) on diabetic neuropathic pain (DNP) mediated by the P2X_7_ receptor in the rat dorsal root ganglia (DRG).

**Results:**

Our results show that NONRATT021972 expression was significantly higher in the DRG of diabetes mellitus (DM) group compared with control group. NONRATT021972 expression in the DRG was reduced when DM rats were treated with NONRATT021972 siRNA. NONRATT021972 siRNA treatment in type 2 DM rats increased the mechanical withdrawal threshold (MWT), the thermal withdrawal latency (TWL) and the sensory nerve conduction velocity (SNCV) of rat tail nerves. After intravenous injection with NONRATT021972 siRNA in DM rats, the P2X_7_, GFAP and TNF-ɑ expression levels in DRG were decreased. An interaction between the RNA (NONRATT021972) and protein (P2X_7_) was predicted by the application of bioinformatics technology. The BzATP-activated currents in DRG non-neurons (satellite glial cells) of DM rats were significantly increased compared to control rats. NONRATT021972 siRNA treatment inhibited the ATP-activated currents in HEK293 cells transfected with pEGFP-P2X_7_.

**Conclusions:**

NONRATT021972 siRNA treatment can decrease the expression levels of P2X_7_ mRNA and protein and inhibit the activation of satellite glial cells (SGCs) in the DRG of type 2 DM rats. Moreover, NONRATT021972 siRNA treatment reduced the release of inflammatory factors (TNF-α), thereby inhibiting the excitability of DRG neurons and reducing mechanical and thermal hyperalgesia in type 2 DM rats.

## Background

Long non-protein-coding RNAs (lncRNAs) are classified as transcripts that are >200 nucleotides (nt) in length [1, 2]. LncRNAs can be transcribed range from 200 bp up to several kilobases in length from either strand and sorted as sense, antisense, bidirectional, intronic, or intergenic based on their nearby protein-coding genes [1–3]. Transcription and post-transcriptional RNA processing, translation, DNA methylation and chromatin architecture are regulated by lncRNAs through local (cis) and long distance (trans) mechanisms [2–4]. LncRNAs can produce a complex regulatory network through interactions with transcription factors, co-activators and/or repressors to influence different aspects of gene transcription [1, 3, 5]. The knockout of some lncRNAs in mice resulted in abnormal functions [3–5]. LncRNAs are also involved in the pathological processes of nervous system diseases [3, 6].

Diabetes mellitus (DM) has become a global epidemic, with an incidence of 11.6 % in our country (representing 113.9 million people). Diabetic neuropathic pain (DNP) is one of the most common chronic complications of diabetes and has typical symptoms of nerve pathological pain, including spontaneous pain, hyperalgesia, and allodynia. Intractable pain induced by diabetes mellitus has become a substantial problem in the field of pain therapy [7]. Fifty percent of diabetic patients suffer from DNP [8]. Studies have shown that people with pre-diabetes are also likely to suffer from neuropathy. Thus, the number of diabetes patients with nerve pathological pain is enormous.

Adenosine triphosphate (ATP) and its analogues bind to P2 receptors [9–11]. P2 receptors can be divided into P2X and P2Y receptors. P2X receptors are ligand-gated ion channels (P2X_1–7_) [9, 10]. Dorsal root ganglia (DRG) transmit sensory signals from the peripheral nerve to the spinal cord [12]. P2X_7_ receptor expressed in satellite glial cells (SGCs) is involved in the pain transmission and the occurrence of neuropathic pain in DM patients [9, 13]. Sensitivities to mechanical pain and thermal pain were significantly decreased in P2X_7_ receptor-knockout mice compared with wild- type mice [13]. Conversely, P2X_7_ receptor expression was increased by inflammatory injury [14]. Furthermore, antagonists of the P2X_7_ receptor could inhibit the pain behavior of neuropathic pain rat [15].

NONRATT021972 is an lncRNA (http://www.noncode.org/show_rna.php?id= NONRATT021972) [16]. At now lncRNA functions are unclear. Our studies showed that the expression levels of lncRNA NONRATT021972 were augmented in the DRG of type 2 DM rat. Therefore, lncRNA NONRATT021972 might participate in the transmission of nociceptive signaling. Inhibiting lncRNA functions in vivo may have therapeutic potential for some diseases [3–5]. This project explored the effects of small interfering RNA (siRNA) treatment against lncRNA NONRATT021972 on the up-regulated expression of P2X_7_ receptor in DRG and the neuropathic pain behaviors in type 2 DM rats. Our work may provide a new experimental basis for the prevention and alleviation of diabetic neuropathic pain.

## Results

### Effects of NONRATT021972 siRNA on NONRATT021972 expression in DRG

**Fig. 1:**
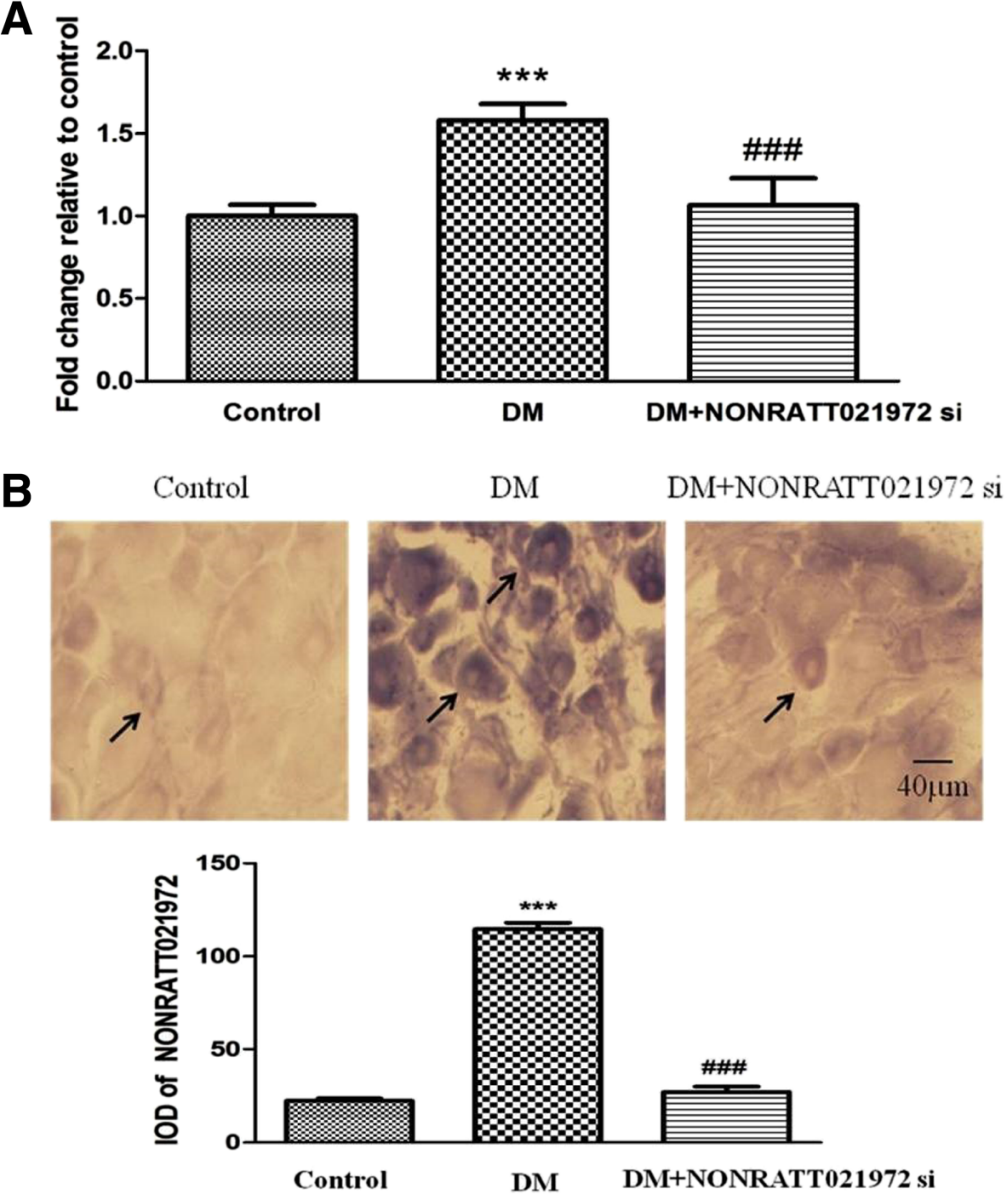
Effects of NONRATT021972 siRNA on NONRATT021972 expression in DRG. **a** The expression of NONRATT021972 in DRG by real-time PCR. The expression of NONRATT021972 in the DM group was significantly higher than that in the control group. Treatment of the DM rats with NONRATT021972 siRNA decreased the expression of NONRATT021972 compared to the DM group. *n* = 6, ^***^ *p* < 0.001 vs control group, ^###^ *p* < 0.001 vs DM group. **b** The expression of NONRATT021972 in DRG evaluated by in situ hybridization (ISH). The expression of NONRATT021972 in DM group was significantly higher than that in the control group, while it was decreased in the DM rats treated with NONRATT021972 siRNA compared with the DM group. The arrows indicate the positive cells in DRG. Scale bar: 40 μm. *n* = 6,^***^ *p* < 0.001 vs control group, ^###^ *p* < 0.001vs DM group

Misexpression of lncRNAs was associated with nervous system diseases. The expression of NONRATT021972 in DRG was evaluated by real-time PCR. The expression of NONRATT021972 in DRG from the DM group was significantly higher than that in the control group (Fig. 1a). Treatment of the DM rats with NONRATT021972 siRNA decreased the expression of NONRATT021972 compared to the DM group (Fig. 1a). The expression of NONRATT021972 in DRG was also evaluated by in situ hybridization(ISH). The expression of NONRATT021972 in the DM group was significantly higher than that in the control group (Fig. 1b). The in situ expression of NONRATT021972 in DRG was also reduced when the DM rats were treated with NONRATT021972 siRNA (Fig. 1b). Our results showed that the expression of NONRATT021972 in the DRG was significantly increased in the DM group than in the control group.

### Effects of NONRATT021972 siRNA on mechanical hyperalgesia and thermal hyperalgesia

**Fig. 2:**
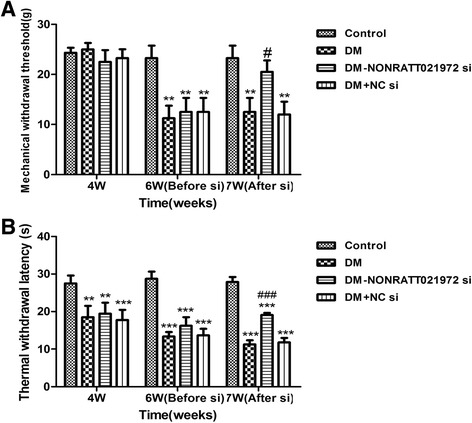
Effects of NONRATT021972 siRNA on the mechanical withdrawal threshold (MWT) and thermal withdrawal latency (TWL) in DM rats. **a** At the 4^th^ week of the experiment, there was no difference in MWT among the groups. At the 6^th^ week of experiment, the MWT in the DM, DM + NONRATT021972 si, and DM + NC si rats was decreased compared to the control rats. There was no significant difference among the DM, DM + NONRATT021972 si, and DM + NC si groups. One week after injection with NONRATT021972 si, the MWL in the DM + NONRATT021972 si group was higher than that in the DM group. *n* = 6,^**^ *p* < 0.01 vs control group, ^#^ *p* < 0.05 vs DM group. **b** At the end of the 4^th^ and 6^th^ weeks of the experiment, the TWL in the DM, DM + NONRATT021972 si, and DM + NC si rats was lower than that in control rats. There was no significant difference among the DM, DM + NONRATT021972 si, and DM + NC si groups. One week after injection with NONRATT021972 si, the MWL in the DM + NONRATT021972 si group was higher than that in the DM group. *n* = 6,^**^ *p* < 0.01, ^***^ *p* < 0.001 vs control group, ^###^ *p* < 0.001vs DM group

Mechanical hyperalgesia and thermal hyperalgesia will be increased during diabetic neuropathic pain state. The effects of the NONRATT021972 si treatment on mechanical hyperalgesia were tested by using von Frey filaments. No difference in the mechanical withdrawal threshold (MWT) was observed among the groups at the 4^th^ week of the experiment (F_(3,20)_ = 1.94, *p* > 0.05). At the 6^th^ week of the experiment, the MWT in the DM, DM + NONRATT021972 si, and DM + NC si rats was lower than that in the control rats (F_(3,20)_ = 9.48, *p* < 0.01). There was no significant difference among the DM, DM + NONRATT021972 si, and DM + NC si groups (F_(2,15)_ = 0.2, *p* > 0.05). One week after injection with NONRATT021972 si, the MWL in the DM + NONRATT021972 si group was higher than that in the DM group (*p* < 0.05) (Fig. 2a).

The effects of the NONRATT021972 si on thermal hyperalgesia were measured by Thermal Paw Stimulation System. At the end of the 4^th^ and 6^th^ weeks of the experiment, the TWL in the DM, DM + NONRATT021972 si, and DM + NC si rats was lower than that in the control rats. There was no significant difference among the DM, DM + NONRATT021972 si, and DM + NC si groups (F_(2,15)_ = 0.90, *p* > 0.05). One week after injection with NONRATT021972 si, the MWL in the DM + NONRATT021972 si group was higher than that in the DM group (*p* < 0.001) (Fig. 2b). The data revealed MWT and TWL in type 2 diabetic model rats were increased after NONRATT021972 siRNA treatment.

### Effects of NONRATT021972 siRNA on sensory nerve conduction velocity

**Fig. 3:**
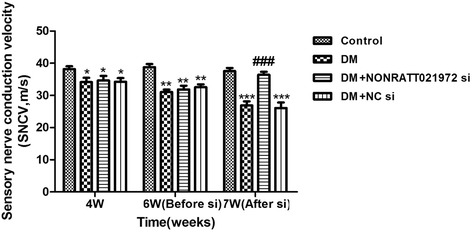
Effect of NONRATT021972 siRNA on the SNCV of the tail nerve in DM rats. At the end of the 4^th^ and 6^th^ weeks of the experiment, the SNCV in DM, DM + NONRATT021972 si, and DM + NC si rats was lower than that in the control rats. There was no significant difference among the DM, DM + NONRATT021972 si, and DM + NC si groups (*p* > 0.05). One week after injection with NONRATT021972 si, the SNCV in DM + NONRATT021972 si group was higher than that in the DM group. *n* = 6,^**^ *p* < 0.01, ^***^ *p* < 0.001 vs control group, ^###^ *p* < 0.001 vs DM group

Measurement of sensory nerve conduction velocity is used to test the function of nerves [17]. At the end of the 4^th^ and 6^th^ weeks of the experiment, the SNCV in the DM, DM + NONRATT021972 si, and DM + NC si rats was lower than that in the control rats (*p* < 0.05). There was no significant difference among the DM, DM + NONRATT021972 si, and DM + NC si groups (*p* > 0.05). One week after injection with NONRATT021972 si, the SNCV in the DM + NONRATT021972 si group was higher than that in the DM group (*p* < 0.05) (Fig. 3). Thus, the SNCV in the DM rats treated with NONRATT021972 siRNA was significantly enhanced compared with the SNCV in the DM rats.

### Effects of NONRATT021972 si on P2X_7_ mRNA and protein expression in the DRG

**Fig. 4:**
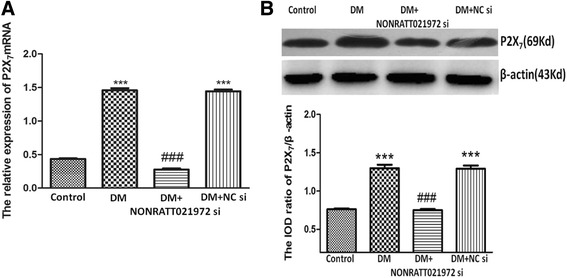
Effects of NONRATT021972 siRNA on P2X_7_ mRNA and protein expression levels in the DRG of DM rats. The optical density values of the P2X_7_ mRNA (**a**) and protein (**b**) expression in the DM group were higher than those in the control group. The expression levels of the P2X_7_ mRNA and protein in the DM + NONRATT021972 si group were lower than those in the DM group. *n* = 6,^***^ *p* < 0.001vs control group, ^###^ *p* < 0.001 vs DM group

P2X_7_ receptor is involved in neuropathic pain. The optical density values (ODV) of P2X_7_ mRNA (Fig. 4a) and protein (Fig. 4b) expression in the four groups were standardized to β-actin. The optical density values of the P2X_7_ mRNA and protein expression in the DM group were higher than those in the control group (*p* < 0.001). The expression levels of the P2X_7_ mRNA and protein in the DM + NONRATT021972 si group were lower than those in the DM group (*p* < 0.001). There was no significant difference between the DM group and the DM + NC si group (*p* > 0.05). Therefore, NONRATT021972 siRNA treatment can influence the upregulation of P2X_7_ receptor in the DM rats.

### Effects of NONRATT021972 siRNA on the coexpression of P2X_7_ and GFAP in DRG based on immunofluorescence

**Fig. 5:**
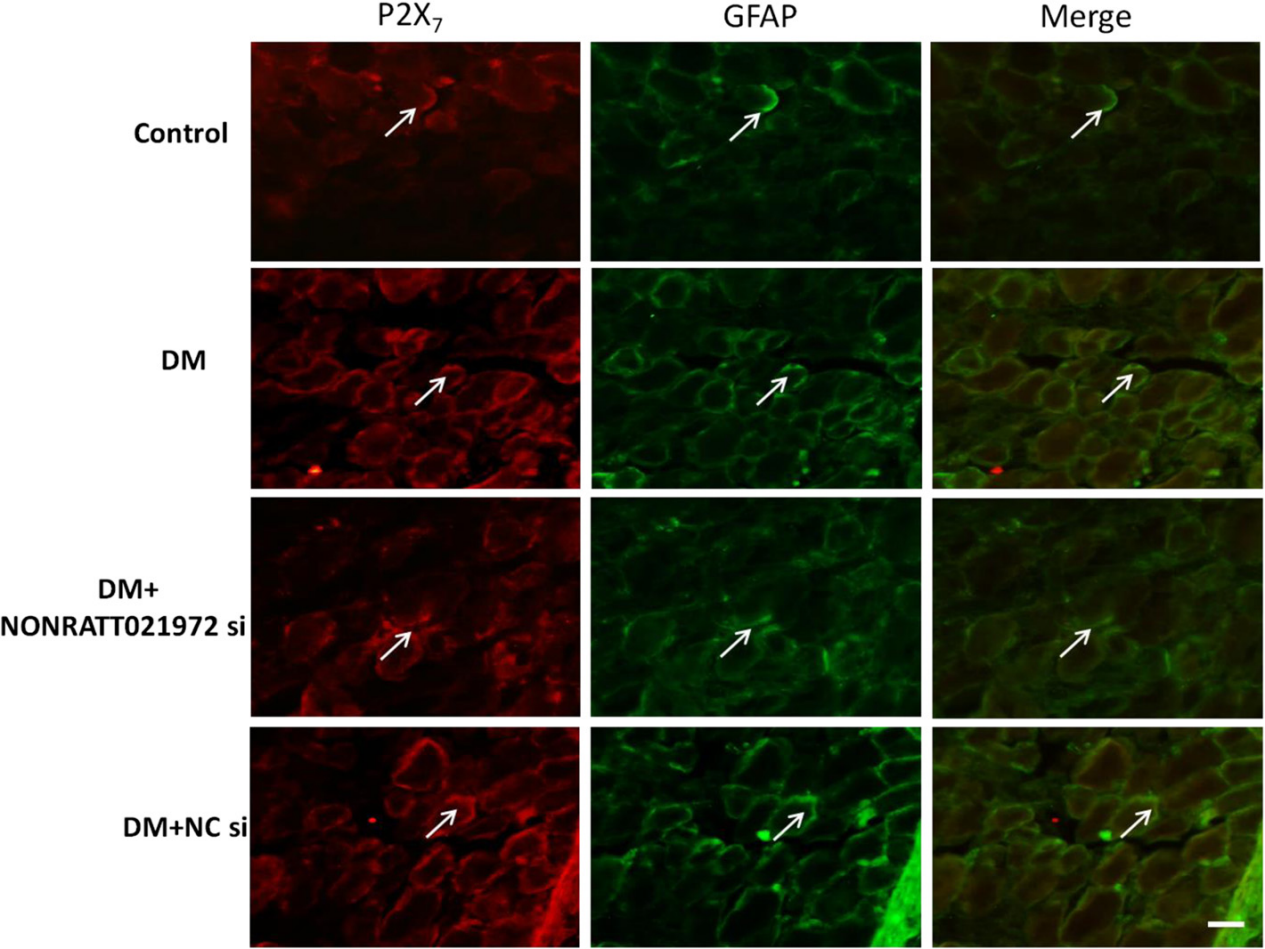
The co-expression values of P2X_7_ and GFAP in the DRG based on double-labeled immunofluorescence. The coexpression of the P2X_7_ receptor with GFAP in the DM group was higher than the values in the control group, whereas the coexpression values in the DM + NONRATT021972 si group were lower than that in DM group. The green signal represents GFAP staining with FITC, and the red signal indicates P2X_7_ staining with TRITC. Merge represents the P2X_7_ and GFAP double staining image. Scale bar: 20 μm

GFAP is the marker of satellite glial cells (SGCs). The up-regulation of GFAP in the DRG suggests the activation of SGCs after nervous injury stimulus [18]. The coexpression values of the P2X_7_ receptor and GFAP in the four groups at the 7^th^ week of the experiment are shown in Fig. 5. P2X_7_ and GFAP were colocalized in the SGCs in DRG. The coexpression values of the P2X_7_ receptor with GFAP in the DM group were higher than that in the control group, whereas the coexpression values in the DM + NONRATT021972 si group were lower than that in the DM group. There was no significant difference between the DM group and the DM + NC si group. NONRATT021972 siRNA treatment may decrease the up-regulation of the P2X_7_ receptor associated with SGC activation in the DRG.

### Effects of NONRATT021972 si on GFAP and TNF-α in DRG

**Fig. 6:**
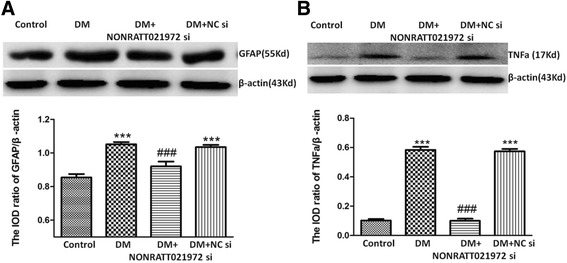
Effects of NONRATT021972 siRNA on GFAP and TNF-α expression in the DRG of DM rats. The expressions of GFAP (**a**) and TNF-α (**b**) in DM group showed a significant increase compared to the control group. The DM + NONRATT021972 si group showed an decrease in the levels of GFAP and TNF-α compared to the DM group. *n* = 6,^***^ *p* < 0.001 vs the control group, ^###^ *p* < 0.001 vs the DM group

TNF-α as an inflammatory factor released from SGCs is involved in the initiation and maintenance of neuropathic pain. The expression values of GFAP (Fig. 6a) and TNF-α (Fig. 6b) in DRG were determined by western blotting. The ODV of GFAP and TNF-α were standardized to β-actin. The expression levels of GFAP and TNF-α in the DM group were higher than that in the control group (*p* < 0.001). The expression levels of GFAP and TNF-α in the DM + NONRATT021972 si group were lower than that in the DM group (*p* < 0.001). There was no significant difference between the DM group and the DM + NC si group (*p* > 0.05). It is possible that NONRATT021972 siRNA treatment decreased the up-regulation of TNF-α and GFAP in DRG and to reduce the pain behaviors in DM rats.

### Predicting the interaction between NONRATT021972 RNA and P2X_7_ receptor

**Fig. 7:**
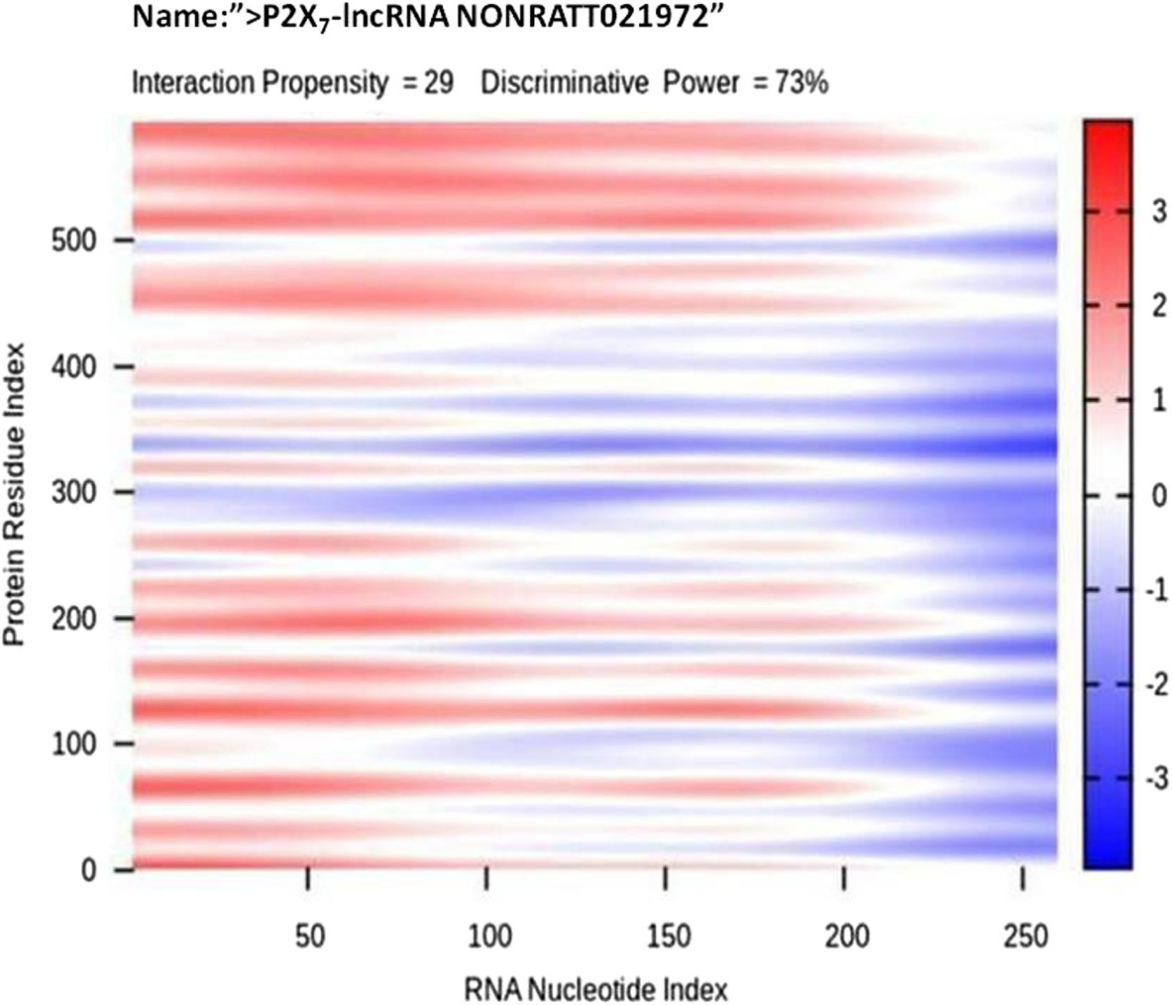
Prediction of the interaction between NONRATT021972 RNA and the P2X_7_ receptor. A high score was obtained for the interaction between NONRATT021972 and the P2X_7_ receptor. The red color represents the area of the interaction

Interactions occur between RNA and proteins [19]. These interactions can be rapidly predicted by the application of bioinformatics technology (CatRAPID) [20]. CatRAPID is an online algorithm that has a predictive function. The method can be used to identify the targets of NONRATT021972 RNA and avoid the blindness of function research. The CatRAPID results revealed a high score for the interaction between NONRATT021972 and P2X_7_ receptor (Fig. 7).

### BzATP-activated currents in DRG non-neuronal cells

**Fig. 8:**
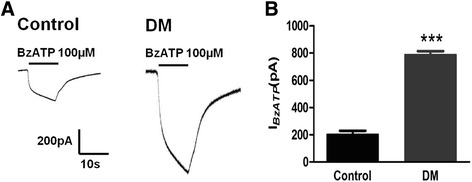
BzATP-activated currents recorded by a whole cell patch clamp. **a** BzATP-activated currents in DRG non-neurons (satellite glial cells) from control and DM rats. The current traces were recorded by the application of 100 μM BzATP. **b** The BzATP-activated current in the DM group was lager than that in the control group. The histogram shows the normalized, mean peak current (mean ± SD, *n* = 12,^***^ *p* < 0.001)

P2X_7_ activation in DRG related to neuropathic pain. P2X_7_ agonist BzATP (100 μM)-activated currents in DRG non-neuronal cells isolated from control rats and DM rats were recorded using a whole cell patch clamp. The results showed that the BzATP-activated current in the DM group was larger than that in the control group (*p* < 0.001) (Fig. 8). Thus, P2X_7_ activation in DRG enhanced the pain behaviors in the DM rats.

### Effects of NONRATT021972 siRNA on the ATP-activated current in HEK293 cells

**Fig. 9:**
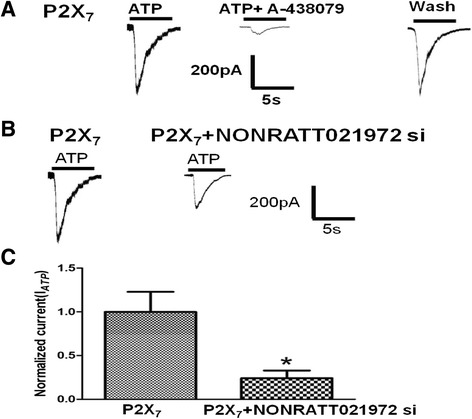
Effects of NONRATT021972 siRNA on the ATP-activated current in HEK293 cells transfected with pEGFP-hP2X_7_ plasmid. NONRATT021972 siRNA reduced the ATP-induced currents in HEK293 cells expressing the hP2X_7_ receptor. **a** Whole cell patch clamp recordings were performed on the HEK293 cells expressing P2X_7_ alone. The plasmid was tagged with EGFP to enable the detection of the transfected cells. Inward currents were evoked by 100 μM ATP at a holding potential of −60 mV. The currents can be inhibited by 10 μM A-438079 (a selective antagonist of the P2X_7_ receptor). **b** ATP activated currents were recorded in the HEK293 cells transfected with P2X_7_ alone or cotransfected with NONRATT021972 siRNA. **c** The histogram shows the normalized, mean peak current (mean ± SD, *n* = 12, ^*^ *p* < 0.05)

We compared the difference of the ATP-activated currents between HEK293 cells transfected with pEGFP-hP2X_7_ plasmid alone or co-transfected with pEGFP-hP2X_7_ plasmid and NONRATT021972 siRNA. The ATP-activated current in HEK293 cells can be blocked by A-438079 (a selective antagonist of the P2X_7_ receptor) (Fig. 9a). The results showed that the ATP-activated currents in the cells transfected with pEGFP-hP2X_7_ plasmid alone were larger than those in the cells co-transfected with pEGFP-hP2X_7_ plasmid and NONRATT021972 siRNA (*p* < 0.05) (Fig. 9b, c). The results support that the NONRATT021972 siRNA treatment reduced pain behaviors in the DM rats by inhibiting the P2X_7_ receptor in DRG.

## Discussion

Many in vitro studies confirmed that lncRNAs have important cellular functions [1, 5]. Experiments using genetic knockout animal models revealed that multiple lncRNAs functioned in disease pathogenesis [3, 6, 21, 22]. Recent studies suggested directions for the development of disease therapies targeting lncRNAs [3, 6, 21, 22]. Our results showed that the expression of NONRATT021972 in the DRG was significantly higher in the DM group than in the control group based on real-time PCR and ISH. The experiments demonstrated that misexpression of lncRNAs was associated with numerous diseases, including nervous system diseases [3, 5, 6]. Our studies also revealed that the expression of NONRATT021972 in DRG from DM rats treated with NONRATT021972 siRNA decreased compared with DM group. Thus, the upregulation of NONRATT021972 in DRG may be associated with the pathological changes that contribute to diabetic neuropathic pain.

Chronic pain is a major public health problem [23–25]. DNP has the typical symptoms of neuropathy pain, including spontaneous pain, hyperalgesia and allodynia [24, 25]. Strategies targeting some lncRNAs have reported inhibitory effects against abnormal cells in vitro and in vivo [26, 27]. The MWT and TWL in type 2 diabetic model rats were lower than those in the control rats in this study. Our experimental results showed that the MWT and TWL in type 2 diabetic rats treated with the NONRATT021972 siRNA in vivo were increased compared with those in the DM rats. Therefore, suppressing NONRATT021972 may relieve neuropathic pain behaviors of type 2 diabetic rats. However, the mechanism underlying the effect of the NONRATT021972 siRNA on the neuropathic pain in type 2 diabetic rats is unknown.

Sensory nerve conduction velocity (SNCV) of the tail nerve is an index of nerve function [17]. SNCV in type 2 DM rats was significantly lower than the control rats, suggesting that there were abnormal changes in the structure and function of the primary sensory afferent nerves. The abnormal changes in the SNCV in DM group were likely to be the pathological basis of diabetic neuropathic pain. Inflammatory injury stimulus in the pathological condition of type 2 DM can affect the SNCV of primary sensory afferent nerves [17]. Additionally, P2X_7_ activation can increase the inflammatory responses [9, 13]. The SNCV in type 2 diabetic rats treated with NONRATT021972 siRNA was significantly increased compared with type 2 diabetic rats. Combined with the down-regulation of the P2X_7_ mRNA and protein expression and GFAP or TNF-α expression in DRG, our results suggested that NONRATT021972 siRNA treatment might reduce the inflammatory injury in DRG sensory afferent nerves. Consequently, NONRATT021972 siRNA treatment may improve the function of primary sensory nerves to decrease the mechanical and thermal hyperalgesia of type 2 DM rats.

DRGs accept the impulse from peripheral nociceptive stimuli and transmit the signal to the central nervous system [12, 28]. The P2X_7_ receptor expressed in DRG is closely associated with pain [9, 13]. We observed that the P2X_7_ mRNA and protein expression levels in the DRG of type 2 DM rats were significantly increased compared with control rats. After treatment of the DM rats with NONRATT021972 siRNA, the expression levels of P2X_7_ mRNA and protein in DRG were decreased compared to DM rats. Thus, NONRATT021972 siRNA treatment could inhibit P2X_7_ receptor to influence the pathological process of DNP.

Primary sensory ganglia, particularly in DRG, contain cell bodies of sensory neurons and satellite glial cells (SGCs) that closely surround the neurons [18, 29]. SGCs can communicate with neurons via signaling molecules [18, 29, 30]. The SGCs in DRG mainly express P2X_7_ receptor [31]. We observed that P2X_7_ receptor was co-expressed with GFAP in SGCs based on immunohistochemistry. The coexpression levels of P2X_7_ and GFAP in DRG were up-regulated in the DM rats compared with the control rats. Notably, GFAP is an SGC marker [18], and nervous system pathological injuries can cause SGC activation [32–34]. In the type 2 DM rats, NONRATT021972 siRNA treatment reduced the up-regulated coexpression values of P2X_7_ and GFAP in DRG. Therefore, the NONRATT021972 siRNA treatment may suppress the up-regulation of the P2X_7_ receptor associated with SGC activation in DRG.

Purinergic signaling involves the interaction between neurons and SGCs in the DRG [32–34]. ATP released from nerve endings can activate the P2X_7_ receptor on the SGCs in DRG. P2X_7_ activation then stimulates SGCs to release cytokines, such as tumor necrosis factor alpha (TNF-α). Inflammatory factors including TNF-α released from SGCs are involved in the initiation and maintenance of neuropathic pain [9–11]. TNF-α can strengthen the excitability of neurons in DRG and enhance its sensitivity to nociceptive stimuli [29, 30, 32]. ATP can activate the P2X_7_ receptor in DRG to mediate the interaction between neurons and SGCs [29, 32, 33]. Our results showed that the expression levels of GFAP and TNF-α in the DM group were higher than those in the control group. After treating the DM rats with NONRATT021972 siRNA, the expression levels of GFAP and TNF-α in the DRG were significantly decreased. Thus, NONRATT021972 siRNA treatment may block P2X_7_ activation in the DRG to inhibit the up-regulation of TNF-α and GFAP and to decrease the pain behaviors of type 2 diabetic rats.

RNA is known to interact with proteins [19]. The application of bioinformatics technology can rapidly predict RNA-protein interactions [20]. We used the CatRAPID online algorithm to evaluate the interaction tendency of NONRATT021972 and a protein (P2X_7_ receptor) based on the secondary structure, hydrogen bonding and molecular inter-atomic forces. This method can help to locate targets of an RNA. Our data showed that the prediction score for the interaction between NONRATT021972 and the P2X_7_ receptor was high. This finding suggests that there is an interaction between NONRATT021972 and the P2X_7_ receptor. The whole cell patch clamp results showed that P2X_7_ agonist BzATP-activated currents in DRG non-neurons (satellite glial cells) from type 2 DM rats were significantly increased compared with control rats. Thus, purinergic signaling was significantly enhanced in the DRG non-neurons (satellite glial cells).

The upregulation of purinergic signaling may increase the release of inflammatory factors from SGCs and promote the excitability of DRG neurons, which increases the pain sensitivity in type 2 DM rats. The results from the HEK293 cell experiment showed that the ATP-activated current in HEK293 cells co-transfected with the pEGFP-hP2X_7_ plasmid and NONRATT021972 siRNA was smaller than that in the cells transfected with the pEGFP-hP2X_7_ plasmid alone. The ATP-activated current in HEK293 cells can be blocked by the P2X_7_ antagonist A-438079. These results revealed that the NONRATT021972 siRNA treatment could relieve pain behaviors in type 2 DM rats by inhibiting the P2X_7_ receptor in DRG.

## Conclusions

In summary, the P2X_7_ receptor in DRG plays an important role in DNP. NONRATT021972 siRNA treatment can decrease the expression levels of P2X_7_ mRNA and protein and the activation of SGCs in the DRG of type 2 DM rats. Furthermore, the NONRATT021972 siRNA treatment reduced the release of inflammatory factors (TNF-α), thereby inhibiting the excitability of DRG neurons and reducing the mechanical and thermal hyperalgesia in type 2 DM rats. These findings highlight the potential of lncRNA NONRATT021972 as a novel therapeutic target in DNP.

## Methods

### Animal model and groups

Zucker diabetic fatty (ZDF) rats (a type 2 diabetic animal model) were purchased from Vital River Laboratories (Beijing, China) at 8 weeks of age and were fed with Purina 5008 diet [35]. Lean ZDF rats without leptin receptor mutations [36], were used as the controls. All the rats were housed in a pathogen-free environment with continuous access to food and water on a 12-h light–dark schedule at 21–25 °C. The animals were cared for following the Medical College of Nanchang University Committee on the Care and Use of Animals guidelines.

Fasting plasma glucose (FPG) and postprandial blood glucose (PBG) of the rats were measured. Blood was collected from the tail vein. An oral glucose tolerance test was performed by administering an oral glucose load (2 g/kg glucose by gavage) and by measuring the blood glucose 2 h after dosing. Diabetes was defined as an FPG ≥7.8 mM or PBG ≥11.1 mM after 6 weeks. Then, the rats were randomly divided into four groups: control group, diabetic model group (DM), DM treated with NONRATT021972 siRNA group (DM + NONRATT021972 si), and DM treated with the scrambled siRNA group (DM + NC si). Each group contained 6 rats. The NONRATT021972 siRNA target sequence was TGTGAATCATGGAAATATC. The NONRATT021972 siRNA and negative control (NC si) were purchased from Invitrogen (Carlsbad, CA, USA). The Entranster^TM^-in vivo transfection reagents were provided by the Engreen Company of Beijing. 320 μl transfection complex consisting of siRNA (NONRATT021972 siRNA or NCsi) was injected into the rats of DM + NONRATT021972 si and DM + NC si groups through sublingual vein according to the reagent’s instructions. Control and DM rats were injected with 320 μl saline. The body weight, blood glucose and behavior of the rats were measured at 1, 4, 6 and 7 weeks after the start of the experiment. After 1 week, the rats were anesthetized with intraperitoneal administration of 50 mg/kg sodium pentobarbital, and the DRG were collected.

### Behavior study

#### Measurement of the mechanical withdrawal threshold (MWT)

Noxious-pressure stimulation was used to evaluate mechanical hyperalgesia. Unrestrained rats were placed inside a clear plastic chamber (22 cm × 12 cm × 22 cm) on a stainless steel mesh floor and allowed to acclimate. Withdrawal responses to mechanical stimulation were determined using von Frey filaments (BME-403, Tianjin) applied through an opening in the stainless-steel mesh floor of the cage (1 cm × 1 cm grid) to an area adjacent to the paw. The filaments were applied in the order of increasing bending force (0.13, 0.20, 0.33, 0.60, 1.30, 3.60, 5.00, 7.30, 9.90, and 20.1 g), with each filament applied 10 times at intervals of 15 s to different parts of the midplantar glabrous skin. The strength of the filaments in the series that evoked at least five positive responses among the ten trials was designated the pain threshold. Values beyond 20.1 g were recorded as 20.1 g.

#### Measurement of thermal withdrawal latency (TWL)

Noxious heat stimulation was applied by the Thermal Paw Stimulation System (BME-410C, Tianjin), and hyperalgesia was determined using thermal stimulation by Hargreaves’ test. The rats were placed in a transparent, square, bottomless acrylic box (22 cm × 12 cm × 22 cm) on a glass plate. After a 30-min habituation period, the plantar surface of their paws was exposed to a beam of radiant heat. Thermal withdrawal latency (TWL) was taken as an index of the nociceptive threshold. The light beam was turned off automatically when a rat lifted its paw, allowing the measurement of time between the beginning of the light beam and the elevation of the foot. This time was designated as the paw withdrawal latency. The hind paws were tested alternately at 5 min intervals. The cut-off time for heat stimulation was 30 s.

#### Measurements of sensory nerve conduction velocity

Rats were anesthetized with 10 % chloral hydrate (300 mg/kg) by intraperitoneal injection. Sensory nerve conduction velocity (SNCV) was evaluated on the tail using platinum electrodes adjacent to the nerve to obtain a recording at the end of 1, 4, 6, and 7 weeks after the experiment. An annular stimulus electrode was located in the distal tail nerve. The negative electrode (black) of the stimulus cathode was located proximal to the tail nerve, whereas the positive electrode (red) was located 1 cm distal from the negative electrode. The recording electrode was 10 cm proximal from the stimulus site. The reference electrode was placed between the stimulus electrode and the recording electrode. The stimulus intensity was fixed to 1.2 mA. SNCV was calculated by the following equation: sensory nerve conduction velocity (m/s) = the distance between recording electrode and stimulus electrode (10 cm) × 10/latency (ms).

### In situ hybridization (ISH)

The rats were anesthetized. The DRG were dissected immediately and fixed in 4 % paraformaldehyde (PFA) for 2 h at room temperature. Then, they were transferred to 15 % sucrose dissolved in 4 % PFA overnight. Tissues were sectioned at 15 mm. Diethyl pyrocarbonate (DEPC) water was used for all of the solutions and appliances of ISH. After deparaffinization and hydration, the slides were digested with 20 μg/ml proteinase K (Promega, Madison) and incubated at 37 °C for 5 min. The slides were stored for 4 h at 42 °C in the pre-hybridization solution in a humidified chamber and then, covered with sense or antisense probes (2.6 ng/μl) that were also denatured (46 °C for 30 min) and incubated at 46 °C for 18 h. After this period, the slides were washed three times with stringency wash solution 1 (50 % formamide, 2X SSC) and 2 (2X SSC) at 46 °C for 5 min. The slides were washed twice in MABT (maleic acid buffer containing Tween 20) for 30 min at 25 °C, transferred to a humidified chamber and covered with 200 μl of blocking buffer added to each section (MABT + 2 % BSA, 1 % blocking reagent (Roche)) for 1 h at 25 °C. Immunodetection was performed using anti-DIG alkaline phosphatase conjugated to antibody (Roche, Basel) diluted in blocking solution (1:1000). After incubation for 1 h in a humidified chamber, the slides were washed five times with MABT for 10 min at 25 °C each wash. The slides were washed twice for 10 min with pre-staining buffer (100 mM Tris pH 9.5, 100 mM NaCl) and incubated in the dark at 37 °C with NBT/BCIP developing solution (Roche, Basel). The reaction was stopped by incubating with PBS (16 h at 4 °C). Finally, the sections were counterstained with Bismarck Brown Y 0.5 % and dehydrated. The slides were mounted with a cover glass with erv-mount resin (Easy Path, São Paulo). Hybridization with sense riboprobes were used as a negative control. The presence of lncRNA in different tissues of the control and DM group was semi-quantified according to the ISH evaluation method proposed by Henke [37].

### Quantitative real-time PCR

Total RNA was isolated from the DRG using the TRIzol Total RNA Reagent (Beijing Tiangen Biotech CO.). The reverse transcription reaction was completed using a RevertAid™ First Strand cDNA Synthesis Kit (Fermentas, Glen Bernie, MD, USA) following the manufacturer’s instructions. The primers were designed with the Primer Express 3.0 software (Applied Biosystems), and the sequences were as follows: NONRATT021972, sense 5'-TAGGATGAGTACCAGTCAGGT-3′; anti-sense 5′-TTTTTGGTTTTTTGACAGGG-3′, and β-actin, sense 5′-GCTCTTTTCCAGCCTTCCTT-3′; anti-sense: 5′-CTTCTGCATCCTGTCAGCAA-3′. P2X_7_, forward 5′-CTTCGGCGTGCGTTTTG-3′, reverse 5′-AGGACAGGGTGGATCCAATG-3′. Quantitative PCR was performed using SYBR® Green MasterMix in an ABI PRISM® 7500 Sequence Detection System (Applied Biosystems, Inc., Foster City, CA, USA). The thermal cycling parameters were 95 °C for 30 s, followed by 40 cycles of amplification at 95 °C for 5 s and 60 °C for 30 s [38]. The amplification specificity was determined by the melting curve, and the results were processed by the software within the ABI7500 PCR instrument.

### Immunohistochemistry

Immunohistochemistry was performed as follows. The rats’ DRG were removed and placed in 4 % paraformaldehyde for 2 h at room temperature. The DRG were washed in 0.1 M PBS prior to incubation in 20 % sucrose in PBS overnight. Then, 12-μm-thick sections were cut using a cryostat (Leica) and mounted onto the slides. The sections were washed in PBS and incubated in blocking solution containing 3 % bovine serum albumin (BSA) in PBS with 0.3 % Triton X-100 for 30 min at room temperature. Primary antibodies against glial fibrillary acidic protein (GFAP) (chicken anti-GFAP, Abcam) and P2X_7_ (rabbit anti-P2X_7_, Abcam) were diluted 1:100 in PBS containing 1 % BSA and incubated overnight at 4 °C. The sections were washed in PBS and incubated with the secondary antibody [goat anti-rabbit TRITC (tetraethyl rhodamine isothiocyanate) (Jackson ImmunoResearch Inc., West Grove, PA, USA) and goat anti-chicken FITC (Beijing Zhongshan Biotech CO.)] diluted 1:200 in PBS for 45 min at 37 °C. Finally, the sections were washed in PBS. Controls omitted the primary antibody. The sections were imaged using a fluorescence microscope (Olympus, Tokyo, Japan). Data were collected from six animals in each group. Five fields randomly selected that contained approximately 50 neurons each were analyzed from each animal and averaged.

### Western blotting

DRG tissues from rats were sampled for western blot analysis. Total protein was extracted by homogenizing the DRG sample by mechanical disruption in lysis buffer (50 mmol/L Tris–Cl, pH 8.0, 150 mmol/L NaCl, 0.1 % sodium dodecyl sulfate (SDS), 1 % Nonidet P-40, 0.02 % sodium deoxycholate, 100 μg/mL phenylmethylsulfonyl fluoride, 1 μg/ml aprofinin, and 1 % protease inhibitor) and incubating on ice for 50 min. The lysates were centrifuged at 12,000 × g for 10 min at 4 °C. The supernatants were collected to measure the protein concentrations using a bicinchoninic acid assay reagent kit and then stored at −20 °C for later use. The supernatants were diluted with sample buffer (250 mmol/L Tris–Cl, 200 mmol/L dithiothreitol, 10 % SDS, 0.5 % bromophenol blue, and 50 % glycerol) and denatured by heating at 95 °C for 10 min. Supernatant samples containing 20 μg protein were loaded onto 10 % SDS-polyacrylamide gels and transferred to polyvinylidene fluoride membranes. The polyvinylidene fluoride membranes were blocked for 1 h at room temperature in 5 % nonfat dried milk in buffer containing 10 mM Tris–HCl (pH 8.0), 150 mM NaCl, and 0.05 % Tween-20. The membranes were incubated with the primary antibodies anti-rabbit P2X_7_ (1:1000, Abcam), anti-chicken GFAP (1:1000, Abcam), or anti-mouse TNF-α (1:1000, Affinity) overnight at 4 °C, followed by incubation with an HRP-conjugated secondary antibody (1:5000, Beijing Zhongshan Biotech CO.) at room temperature. The membranes were stripped and incubated with mouse anti-β-actin (Sigma-Aldrich) to verify equal loading of the proteins in the western blot analysis. The densities of the bands were determined using Image J Software.

### Rapid prediction of the interaction between RNA and protein

Bioinformatics technology can be applied to rapidly predict the interaction between RNA and proteins (fast predictions of RNA and protein interactions and domains, CatRAPID) [20]. We used the CatRAPID online algorithm forces to evaluate the interaction of the NONRATT021972 RNA and the protein (P2X_7_ receptor) based on the secondary structure, hydrogen bonding and molecular inter-atomic forces. This method can be used to locate RNA targets.

### Isolation of DRG non-neurons

The rats were anesthetized with urethane (1.2 g/kg, intraperitoneally, i.p.). The DRG were isolated from the rats and transferred immediately into Dulbecco’s modified Eagle’s medium (DMEM, Sigma) at pH 7.4 and 340 mosmol/kg. After removal of the attached nerves and surrounding connective tissues, the DRGs were minced with dissecting spring scissors and incubated with trypsin (0.5 mg/ml; type III, Sigma), collagenase (1.0 mg/ml; type IA, sigma) and DNase (0.1 mg/ml; type IV, Sigma) in 5 ml of DMEM at 35 °C in a shaking bath for 35–40 min. Then, soybean trypsin inhibitor (1.25 mg/ml; type II-S, Sigma) was added to stop the enzymatic digestion. The isolated non-neuronal cells (satellite glial cells) were transferred into a 35-mm culture dish and kept stationary for 30 min [39]. The experiments were performed at room temperature (20–30 °C).

### HEK 293 cell culture and transfection

HEK 293 cells were grown in Dulbecco’s modified Eagle’s medium supplemented with 10 % fetal bovine serum and 1 % penicillin and streptomycin at 37 °C in a humidified atmosphere containing 5 % CO_2_. The cells were transiently transfected with the human pcDNA3.0-EGFP-P2X_7_ plasmid and NONRATT021972 siRNA using the Lipofectamine 2000 reagent (Invitrogen) according to the manufacturer’s instructions. When the HEK293 cells were 70–80 % confluent, the cell culture medium was replaced with OptiMEM two hours prior to transfection. The transfection media were prepared as follows: (a) 4 μg of DNA or siRNA diluted into 250 μl final volume of OptiMEM; (b) 10 μl of Lipofectamine2000 diluted into 250 μl final volume of OptiMEM; and (c) the Lipofectamine-containing solution was mixed with the plasmid-containing solution and incubated at RT for 20 min. Subsequently, 500 μl of the cDNA/Lipofectamine complex solution was added to each well. The cells were incubated for 6 h at 37 °C in 5 % CO_2_. After incubation, the cells were washed in MEM containing 10 % FBS and incubated for 24–48 h. The GFP fluorescence was evaluated as a reporter for the transfection efficiency. Whole-cell patch clamp recordings were performed 1–2 days after transfection [40].

### Electrophysiological recordings

Electrophysiological recording was performed using a patch/whole cell clamp amplifier (Axopatch 200B) [39]. A micropipette was filled with an internal solution (in mM) containing KCl (140), MgCl_2_ (2), HEPES (10), EGTA (11), and ATP (5); the solution’s osmolarity was adjusted to 340 mosmol/kg with sucrose and its pH was adjusted to 7.4 with KOH. The external solution (in mM) contained NaCl (150), KCl (5), CaCl_2_ (2.5), MgCl_2_ (1), HEPES (10), and D-glucose (10); the solution’s osmolarity was adjusted to 340 mosmol/kg with sucrose, and its pH was adjusted to 7.4 with NaOH. The resistance of the recording electrodes was in the range of 1 to 4 MΩ, with 3 MΩ being optimal. A small patch of membrane underneath the tip of the pipette was aspirated to form a seal (1–10 GΩ), and then more negative pressure was applied to rupture it to establish a whole-cell mode. The holding potential (HP) was set to −60 mV. A-438079 (a selective antagonist of P2X_7_ receptor, Tocris Bioscience, Ellis-ville, MO), BzATP, and ATP (Sigma Company) were dissolved in the external solution and delivered by gravity flow from an array of tubules (500 μm O.D., 200 μm I.D.) connected to a series of independent reservoirs. The distance from the tubule mouth to the examined cell was approximately 100 μm. Rapid solution exchange was achieved by shifting the tubules horizontally with a micromanipulator.

### Statistical analysis

Significant differences were evaluated using one-way ANOVA followed by Dunnett’s or Tukey’s tests. The statistical analyses were performed using GraphPad Prism version 5.0. The results were expressed as the mean ± SD. to show variation between groups. Differences were considered significant when p˂0 .05.
